# Formation and function of multiciliated cells

**DOI:** 10.1083/jcb.202307150

**Published:** 2023-11-30

**Authors:** Qian Lyu, Qingchao Li, Jun Zhou, Huijie Zhao

**Affiliations:** 1 Center for Cell Structure and Function, Shandong Provincial Key Laboratory of Animal Resistance Biology, Collaborative Innovation Center of Cell Biology in Universities of Shandong, College of Life Sciences, Shandong Normal University, Jinan, China; 2 State Key Laboratory of Medicinal Chemical Biology, Haihe Laboratory of Cell Ecosystem, College of Life Sciences, Nankai University, Tianjin, China

## Abstract

In vertebrates, multiciliated cells (MCCs) are terminally differentiated cells that line the airway tracts, brain ventricles, and reproductive ducts. Each MCC contains dozens to hundreds of motile cilia that beat in a synchronized manner to drive fluid flow across epithelia, the dysfunction of which is associated with a group of human diseases referred to as motile ciliopathies, such as primary cilia dyskinesia. Given the dynamic and complex process of multiciliogenesis, the biological events essential for forming multiple motile cilia are comparatively unelucidated. Thanks to advancements in genetic tools, omics technologies, and structural biology, significant progress has been achieved in the past decade in understanding the molecular mechanism underlying the regulation of multiple motile cilia formation. In this review, we discuss recent studies with ex vivo culture MCC and animal models, summarize current knowledge of multiciliogenesis, and particularly highlight recent advances and their implications.

## Teaser

Lyu et al. summarize the current knowledge of multiciliogenesis by which the respiratory tract, brain ventricles, and reproductive tracts of vertebrates form multiple motile cilia for tissue homeostasis.

## Introduction

Cilia are microtubule-based hair-like structures that protrude from the cell surface. Each cilium comprises a basal body (BB) converted from a mature centriole, a centriolar microtubule-extended axoneme, and a ciliary membrane. The BB anchors the cilium to the plasma membrane, and the axoneme coordinates with the ciliary membrane to execute ciliary functions.

Based on their motility, cilia can be categorized into non-motile and motile cilia. Non-motile cilia are commonly termed primary cilia or sensory cilia and function as a signaling hub to transduce external stimuli into cellular responses (Goetz and Anderson, 2010). Most mammalian cells possess a singular non-motile cilium except for olfactory neurons and choroid plexus epithelial cells, which contain multiple non-motile cilia (Falk et al., 2015; Narita and Takeda, 2015). By contrast, motile cilia can move and are present in large numbers on terminally differentiated cells (multiciliated cells, MCCs) in epithelial tissues of the brain ependyma, airway, and reproductive ducts. Motile cilia also occur as solitary structures such as sperm flagella and embryonic node cilia (Jain et al., 2010). The beating of motile cilia creates fluid flow on the cell surface to expel mucus or power cellular movements (Del Bigio, 2010; Dirksen, 1971; Sorokin, 1968). In addition to the well-appreciated role in motility, motile cilia of airway epithelia can perform sensory functions like primary cilia (Mao et al., 2018; Nordgren et al., 2014; Shah et al., 2009) and also facilitate SARS-CoV-2 infection in airway epithelia (Wu et al., 2023).

Structurally, in contrast to the typical 9+0 axoneme arrangement of primary cilia, most motile cilia display a 9+2 axoneme arrangement with nine peripheral doublet microtubules (DMTs) surrounding the central apparatus (CA) that consists of a central pair of microtubules (CP) and CP-associated projections (Spassky and Meunier, 2017; Zhao et al., 2020b). The DMTs are decorated with motility related structures, including axonemal dyneins (outer dynein arm [ODA] and inner dynein arm [IDA] complexes), radial spokes (RSs), and nexin–dynein regulatory complexes (N-DRCs; Ishikawa, 2017). Besides the typical 9+2 motile cilia, the motile nodal cilia in the embryonic ventral node have a 9+0 axoneme structure and the motile cilia on the rabbit embryonic notochordal plate display a 9+4 arrangement (Feistel and Blum, 2006; Nonaka et al., 1998).

In primary ciliated cells, cilia are assembled through a complex multistep process referred to as ciliogenesis, which has been extensively reviewed elsewhere (Ishikawa and Marshall, 2011; Santos and Reiter, 2008; Zhao et al., 2023a). The corresponding process of multiple motile cilia formation in MCCs is termed multiciliogenesis, which follows MCC fate determination and generally includes centriole amplification, migration and docking, and axoneme assembly. Recent advances in imaging, structural biology, genomics, and proteomics have revealed significant insights into multiciliogenesis, especially in mammalian brain and airway epithelial MCCs. Here, we discuss the results of recent studies conducted in ex vivo MCCs and animal models, present an overview of the proteins and associating networks that regulate the formation of multiple motile cilia, and focus on recent progress in the process of multiciliogenesis. Along with a synthesis of our current knowledge regarding the molecular mechanisms of multiciliogenesis, we present potential routes for future studies.

### MCC cell fate acquisition and the transcriptional control of multiciliogenesis

The extrinsic cues and intrinsic properties that determine the specification of MCC cell fate during development have been extensively studied. MCC progenitor cells respond to particular environmental cues by stimulating the expression of MCC-specific genes. In this section, we highlight the signaling pathways that lead to MCC differentiation and summarize associated regulators that govern the MCC-specific gene expression (Table S1).

#### Signaling pathways

MCCs in the brain ventricles arise primarily during development and persist for many years. Although radial glia and subventricular zone astrocytes possess the ability to generate new ependymal MCCs (Luo et al., 2008; Spassky et al., 2005), limited replacement of ventricular MCCs only occurs under pathological conditions or in aging mice (Luo et al., 2008). Meanwhile, MCCs in the airway and reproductive organs can regenerate continuously throughout life (Kessler et al., 2015; Rock et al., 2009; Spassky and Meunier, 2017). The airway epithelium contains multiple cell types such as multiciliated, basal, neuroendocrine, and club cells (previously named Clara cells). Lineage tracing analysis suggests that basal and club cells can differentiate into MCCs (Rawlins et al., 2009; Ruysseveldt et al., 2021). Single-cell transcriptomics of *Xenopus* organoids has recently revealed that basal cell differentiation requires a multipotent “early epithelial progenitor” (Lee et al., 2023). During MCC differentiation, different cell types exclusively express distinct markers. Undifferentiated stem cells (SOX2 and/or SOX3) undergo maturation to generate epithelial progenitors (HAS1), which further differentiate into basal cells (P63). The basal cells can form multiple cell types such as MCCs (FOXJ1) during embryonic development and act as adult stem cells to replenish MCCs upon regeneration (Daniely et al., 2004; Lee et al., 2012, 2023; Liu et al., 2013).

In general, the differentiation of progenitor cells into MCC precursor cells requires the crosstalk of multiple signaling pathways. The bone morphogenetic protein (BMP) and Notch signaling pathways are both involved in the MCC cell fate determination (Cibois et al., 2015; Deblandre et al., 1999; Liu et al., 2007; Ma and Jiang, 2007; Marcet et al., 2011b; Morimoto et al., 2010; Omiya et al., 2021). Inhibited Notch signaling promotes MCC differentiation, but constitutive activation conversely decreases the MCC population (Lewis and Stracker, 2021). While Notch receptors (NOTCH1/2/3/4) and their ligands (Delta-like and Jagged: DLL1/3/4, and JAG1/2) coordinate differentiation of multiple cell types during lung development, Jagged family ligands may exclusively regulate the differentiation program that produces MCCs (Stupnikov et al., 2019). Similarly, the BMP signaling pathway negatively regulates the differentiation of progenitor cells into MCCs. Overactivation of BMP signaling decreases the number of MCCs, while BMP inhibition promotes the MCC differentiation in *Xenopus* epidermal, mouse ventricular, and human airway epithelia (Cibois et al., 2015; Nishimura et al., 2010; Omiya et al., 2021). In addition, inhibitory Smad proteins negatively regulate the canonical SMAD2-dependent transforming growth factor-β (TGF-β) signaling pathway, which however is not required for the MCC cell fate determination but essential for the ciliary length control (Tözser et al., 2015). Moreover, the Hippo pathway also plays a role in MCC differentiation via its transcriptional coactivator YAP. Disruption of YAP in airway epithelial progenitors prevents multiciliogenesis (Mahoney et al., 2014; van Soldt and Cardoso, 2020; van Soldt et al., 2019). Recent studies also demonstrate a requirement of the dynamically modulated Wnt/β-catenin signaling in cell fate specification of multiciliated and secretory cells (Brechbuhl et al., 2011; Hou et al., 2019; Huang and Niehrs, 2014; Malleske et al., 2018; Schmid et al., 2017). In basal cells, Wnt/β-catenin signaling activates P63, which promotes basal cell proliferation and prevents its differentiation into MCCs. However, in MCCs, Wnt/β-catenin signaling positively regulates the expression of FOXJ1, a master transcriptional factor for multiciliogenesis. Disturbance of Wnt/β-catenin signaling thus also inhibits MCC differentiation (Haas et al., 2019).

#### Gene expression regulators

**Figure 1.**
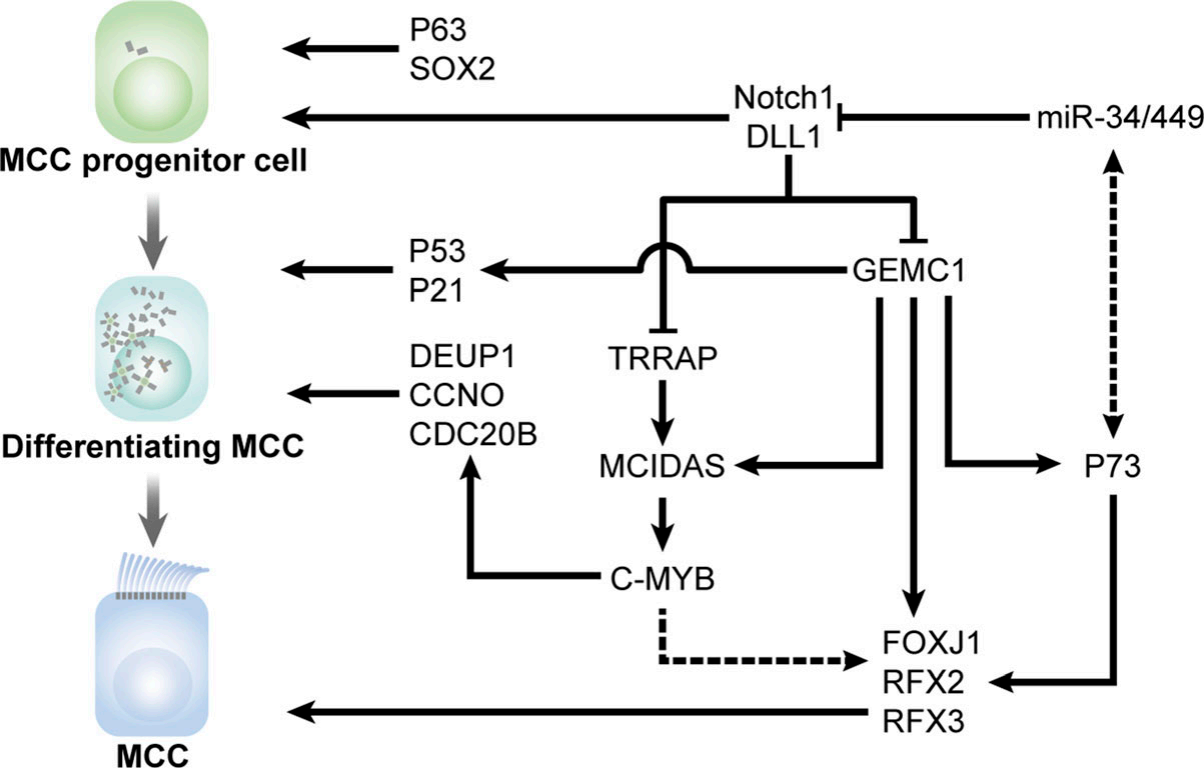
**Regulation of MCC cell fate determination.** The Notch signaling pathway inhibits the differentiation of MCC progenitor cells that express P63 and SOX2. Upon the activation of MCC differentiation, miR-34/449 can inhibit the Notch signaling pathway, which leads to the expression of key regulators, including TRRAP, GEMC1, and MCIDAS. GEMC1 induces cell cycle exit through the P53-P21 pathway and acts upstream of MCIDAS and C-MYB to control the expression of centriole amplification-related regulators such as DEUP1, CCNO, and CDC20B. On the other hand, GEMC1, C-MYB, and P73 can regulate the expression of motile cilia-related master regulators such as FOXJ1, RFX2, and RFX3.

MicroRNAs are a group of small non-coding RNAs regulating gene expression at the posttranscriptional level. The miR-34/449 microRNA family is involved in multiciliogenesis, as miR-34/449 deficient mice exhibit fertility and respiratory dysfunction (Lizé et al., 2010; Marcet et al., 2011a, 2011b; Song et al., 2014; Wu et al., 2014; Yuan et al., 2019). The miR-34/449 family contains six homologous miRNAs (miR-34a/b/c and miR-449a/b/c), which redundantly regulate MCC cell fate determination by targeting Notch signaling (Marcet et al., 2011a, 2011b). Consistently, a significant reduction in FOXJ1-positive cells is seen in the efferent ductular epithelia of miR-34/449 deficient mice (Wu et al., 2021). These studies demonstrate the crucial role of the miR-34/449 family in MCC cell fate acquisition (Fig. 1).

Geminin coiled-coil domain-containing protein 1 (GEMC1) and Multicilin (also known as MCIDAS) are the most upstream transcription regulators specifically required for MCC differentiation. Upon inhibition of Notch signaling by the miR-34/449 microRNAs, the expression of GEMC1 and MCIDAS is activated, indicating that both transcription regulators function as downstream effectors of the Notch signaling pathway (Arbi et al., 2016; Ma et al., 2014; Stubbs et al., 2012; Terré et al., 2016). GEMC1 acts upstream of MCIDAS since GEMC1 depletion prohibits the MCIDAS expression in zebrafish kidney tubules (Zhou et al., 2015). GEMC1 directly regulates the expression of several transcription regulators critical for MCC differentiation, including MCIDAS, FOXJ1, RFX2, and RFX3, whereas MCIDAS governs the expression of genes required for centriole amplification, including *Ccno*, *Cdc20b*, and *Deup1* (Fig. 1; Lu et al., 2019). In addition, MCIDAS regulates the expression of C-MYB, which is required for centriole amplification during multiciliogenesis (Fig. 1; Tan et al., 2013). C-MYB has been proposed to function upstream of FOXJ1 (Pan et al., 2014; Tan et al., 2013), although the expression of FOXJ1 is surprisingly unaffected in *Mcidas* mutant MCCs (Lu et al., 2019).

Due to the lack of a DNA binding domain, MCIDAS is reported to associate with E2F4 or E2F5 transcription factors and their cofactor DP1 to form the E2F4/E2F5-DP1-MCIDAS (EDM) complex, which cooperates to promote the transcription of essential genes required for multiciliogenesis (Ma et al., 2014). Despite the structural similarities between GEMC1 and MCIDAS, GEMC1 preferably interacts with E2F5 via its distinct C-terminal domain (Arbi et al., 2016; Lu et al., 2019). Consistently, E2F4 and E2F5 have also been linked to MCC differentiation in the murine brain, airway, and germline (Danielian et al., 2007, 2016; Lindeman et al., 1998; Ma et al., 2014). In addition to E2F4 and E2F5, a proximal proteomic analysis revealed distinct interactions with SWI/SNF subcomplexes: GEMC1 and MCIDAS substantially interact with the ARID1A-containing BAF complex and the BRD9 containing ncBAF complex, respectively, interactions required for the transcriptional activity of GEMC1 and MCIDAS during MCC formation (Lewis et al., 2023). Moreover, the transformation/transcription domain-associated protein TRRAP acts downstream of the Notch signaling to regulate the transcription of *Mcidas* and many other MCC genes (Fig. 1; Wang et al., 2018). It is interesting as TRRAP can also not bind DNA and both TRRAP and MCIDAS that are needed to form a complex with the E2F family proteins for transcriptional activity (Ma et al., 2014; Wang et al., 2018). Future studies are needed to explore how TRRAP, E2F, and MCIDAS are linked together to control MCC differentiation.

P53 family transcription factors also regulate multiciliogenesis. As aforementioned, P63 is essential for the identity and maintenance of epithelial basal progenitor cells (Fig. 1). Loss of P63 leads to severe epidermal and craniofacial abnormalities in mice and impairs the differentiation of multipotent cells into airway epithelial cells (Bilodeau et al., 2021; Yang et al., 1999). P73, another P53 family transcription factor, has been linked to MCC differentiation as a direct regulator of motile cilia–associated genes such as *Foxj1*, *Rfx2*, and *Rfx3* (Fig. 1; Marshall et al., 2016; Nemajerova et al., 2016). The *p73*-deficient mice exhibit congenital hydrocephalus, sterility, chronic lung inflammation, and sinus (Yang et al., 2000). While P73 is essential for multiciliogenesis in the airway and reproductive ducts (Marshall et al., 2016; Nemajerova et al., 2016; Wildung et al., 2019), the function of P73 in ventricular multiciliogenesis is compensated by the miR-449a/b/c microRNAs (Wildung et al., 2019), indicating tissue-specific molecular circuits for MCC cell fate determination (Fig. 1). Moreover, the signal of P73 was gone in *Gemc1* knockout MCCs but steadily existed in *Mcidas* knockout cells (Terré et al., 2019), suggesting *p73* as a downstream target gene of GEMC1 (Fig. 1). The P53-P21 pathway also functions downstream of GEMC1 to induce cell cycle arrest, allowing ependymal progenitors to differentiate into MCCs (Fig. 1; Ortiz-Álvarez et al., 2022).

FOXJ1 and RFX family transcription factors are important for controlling ciliary gene expression (Fig. 1). FOXJ1, a forkhead domain-containing transcription factor, is exclusively necessary for motile cilia formation. In vertebrates, FOXJ1 is highly expressed in multiciliated tissues. FOXJ1 loss of function causes left-asymmetry defects and hydrocephalus in mice and humans (Brody et al., 2000; Hou et al., 2023; Shapiro et al., 2021; Wallmeier et al., 2019). Leucine-rich repeat-containing protein 6 (LRRC6) can regulate the nuclear translocation of FOXJ1. The absence of LRRC6 causes the mislocalization of FOXJ1 in the cytoplasm, downregulating the transcriptional levels of motile cilia–related genes (Kim et al., 2023). As for RFX transcription factors, they play roles in both primary ciliogenesis and multiciliogenesis by regulating a set of proteins involved in ciliary transport and BB anchoring (Thomas et al., 2010). RFX2 and RFX3 appear to function redundantly in the control of multiciliogenesis in mice ependymal cells (Lemeille et al., 2020). Interestingly, FOXJ1 and RFX3 have been proposed to regulate the expression of ciliary proteins cooperatively (Didon et al., 2013).

### Multiple motile cilia formation

Once the MCC cell fate is determined, these cells have to exit the cell cycle and create a permissive environment for massive centriole production, a process involving multiple cell cycle regulators and MCC-specific cellular structures, including fibrogranular materials and deuterosomes (Levine and Holland, 2017; Sorokin, 1968). The newly nucleated centrioles sequentially undergo migration to the apical surface, docking to the plasm membrane, and eventually templating the ciliary axoneme. In this section, we will introduce the current knowledge on these cellular events, discuss the controversies in the field, and particularly focus on recent advances in understanding axoneme assembly.

#### Centriole amplification

A key feature of MCC is that each cell contains multiple motile cilia extending from the apical surface. However, MCC precursor cells only possess a centrosome consisting of two parental centrioles (a mature mother centriole and an immature daughter centriole). Intriguingly, electron microscopy imaging of differentiating MCCs has revealed that upon the initiation of MCC differentiation, each parental centriole can produce more than one procentriole, and the majority of the total centrioles are nucleated via unique cytoplasmic organelles called deuterosomes (Mahjoub et al., 2022; Sorokin, 1968; Yan et al., 2016). Therefore, this raises questions regarding how the MCC precursor cells gain the ability to break the strict centriole-duplication rule that only one new centriole is produced per parental centriole and how those cells generate the specific deuterosome organelle for massive centriole amplification.

**Figure 2.**
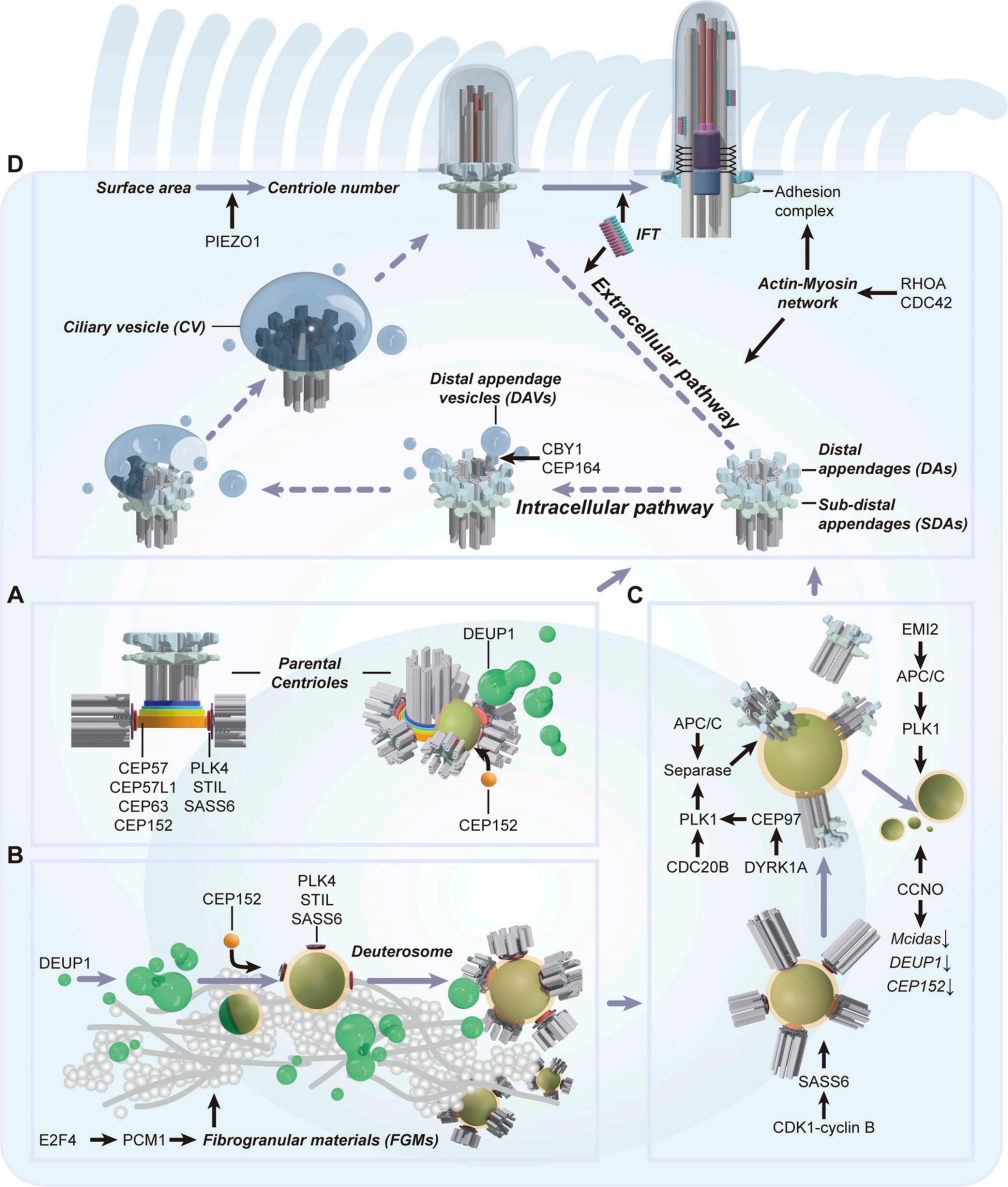
**Formation of multiple motile cilia. (A)** Parental centriole dependent centriole amplification. The CEP57–CEP63–CEP152 cascade mediates the initiation of parental centriole-dependent centriole amplification. In differentiating MCCs, both parental centrioles can generate multiple procentrioles. Note that the daughter centriole of parental centrioles can accumulate DEUP1 and promote the deuterosome assembly. **(B)** Deuterosome-dependent centriole amplification. DEUP1 and CEP152 mediate the initiation of deuterosome-dependent centriole amplification. PCM1 and other fibrogranular material (FGM) components form fibrous granules, which can similarly enrich DEUP1. Regional concentrated DEUP1 can self-assemble into macromolecular deuterosomes. Both centriole amplification pathways utilize common downstream regulators such as PLK4, SASS6, and STIL to generate procentrioles. **(C)** Centriole dissociation and deuterosome disassembly. As procentrioles grow and mature, CDK1-cyclin B phosphorylates SASS6, which in turn destabilizes the cartwheel structure, and Separase cooperates with other regulators such as PLK1 and CDC20B to release the maturing centrioles from their nucleating platforms, including parental centrioles and deuterosomes. Meanwhile, CCNO downregulates the expression of centriole amplification-related genes and collaborates with EMI2 and PLK1 to accomplish the deuterosome disassembly or clearance. **(D)** Assembly of multiple motile cilia. As centrioles gain the distal and subdistal appendages (DAs and SDAs), they conduct polarized migration with the help of IFT and the actin–myosin network. MCCs may adopt either extracellular or intracellular pathways to form motile cilia, although CBY1-mediated distal appendage vesicle (DAV) accumulation is involved in multicilia formation. Once docked to the plasm membrane, basal bodies converted from mature centrioles are fastened to the actin network by a ciliary adhesion complex. The centriole number in MCCs is calibrated to the apical surface area via PIEZO1.

In most cycling cells, new centrioles are assembled in the proximal vicinity of the parental centrioles through stepwise recruitment of a series of protein complexes (Loncarek and Bettencourt-Dias, 2018; Nigg and Holland, 2018; Vasquez-Limeta and Loncarek, 2021). Based on the current literature, CEP57 (centrosomal protein of 57 kD), which localizes to the centriole via binding centriolar microtubules, functionally cooperates with its paralog CEP57L1 to recruit CEP63, which interacts with CEP152 to ensure its centriolar loading (Brown et al., 2013; Lukinavičius et al., 2013; Zhao et al., 2020a). Then CEP152 interacts with the serine/threonine-protein kinase PLK4, a master regulator of centriole biogenesis (Hatch et al., 2010), which further recruits CPAP, STIL, SASS6, and other proteins to initiate the procentriole assembly (Fig. 2 A; Cizmecioglu et al., 2010; Moyer et al., 2015; Tang et al., 2011). It remains unclear how the parental centrioles in MCC precursor cells generate multiple procentrioles. In cycling cells, the control of centriole numbers depends on the protein levels of key duplication proteins such as PLK4, SASS6, and STIL (Bauer et al., 2016). Indeed, overexpression of either key centriole duplication protein can induce the production of random supernumerary centrioles (Arquint et al., 2012; Bettencourt-Dias et al., 2005; Strnad et al., 2007). Consistently, the expression of these factors is strikingly elevated in MCCs undergoing centriole amplification compared with the low abundance in proliferating cells (Kim et al., 2018; Zhao et al., 2013). Thus, it is plausible that the ability of parental centrioles to generate multiple new centrioles is attributed to the highly elevated protein levels of these key duplication factors.

During MCC differentiation, about 90% of newly assembled centrioles are produced by the deuterosome structure, which is formed through DEUP1 oligomerization or condensation (Yamamoto et al., 2021; Zhao et al., 2013). DEUP1, like its paralogue CEP63, can interact with CEP152 and trigger the downstream cascade of canonical centriole biogenesis at the deuterosome (Fig. 2 B; Zhao et al., 2013). Based on the observation of deuterosome preferably associating with the daughter centriole, it has been proposed as the primary nucleation site for deuterosome formation and centriole amplification (Al Jord et al., 2014). Recent studies reveal that in the absence of parental centrioles, deuterosomes can form normally in the cytosol of MCCs (Mercey et al., 2019a; Nanjundappa et al., 2019; Zhao et al., 2019), indicating that the daughter centriole is dispensable for deuterosome formation. The unique accumulation of DEUP1 at the daughter centriole increases the local concentration of DEUP1, which further promotes its self-assembly into macromolecular deuterosomes in the vicinity of the daughter centriole (Fig. 2 B). Similarly, fibrogranular materials (FGMs) in MCCs have been proposed to be deuterosome precursors (Anderson and Brenner, 1971; Dirksen, 1971; Sorokin, 1968). However, after the disruption of FGMs by depleting PCM1, a core FGM component, deuterosomes are still formed but extensively dispersed in the cytosol, and the deuterosome size is significantly reduced (Zhao et al., 2021), suggesting that FGMs function in the regional enrichment of deuterosome components as well (Fig. 2 B). Analysis of *Pcm1* knockout mice further reveals that the lack of PCM1 does not affect the number of centrioles but causes a delay in centriole biogenesis (Hall et al., 2023). These findings indicate that the daughter centriole and the FGMs may enrich the local deuterosome proteins, greatly facilitating deuterosome assembly (Fig. 2 B).

Interestingly, the parental centriole-dependent and the deuterosome-dependent centriole amplification pathways are interconnected. In mouse tracheal MCCs in vitro, transient depletion of DEUP1 enhances CEP63-mediated parental centriole-dependent centriole amplification and causes ciliogenesis defects in the early stage of multiciliogenesis (Zhao et al., 2013). Further analysis of the whole process of centriole amplification in DEUP1-depleted MCCs reveals a dramatic compensation for the loss of DEUP1 from the parental centriole-dependent pathway. In DEUP1-depleted MCCs, each parental centriole can nucleate tens of procentrioles in the early stage of centriole amplification, and those newly formed procentrioles/centrioles can further template the second round of centriole duplication. In addition, the ciliogenesis defects can be rescued in the later stage of multiciliogenesis, suggesting that the lack of DEUP1 causes a delay in centriole biogenesis. However, MCCs of the *Deup1* and *Cep63* double-mutant mice can generate comparable centrioles to the wild-type MCCs (Mercey et al., 2019b). This finding is interesting regarding the difference between the in vitro and in vivo systems. Future work will be needed to investigate how these centrioles are nucleated in the absence of DEUP1 and CEP63, and whether/how the centriole duplication proteins such as CEP152 are involved in centriole amplification in *Deup1* and *Cep63* double-mutant MCCs.

In addition to the core component DEUP1, other structural proteins relative to the deuterosome have been identified in the past decade, including E2F4, CCDC78, and CDC20B. The transcription factor E2F4 not only transcriptionally regulates the expression of DEUP1 but also forms the cytoplasmic E2F4-PCM1 granules for deuterosome assembly (Mori et al., 2017). CCDC78 is reported to mediate the deuterosome distribution of CEP152 and is essential for centriole amplification in *Xenopus* epidermal MCCs (Klos Dehring et al., 2013). CDC20B localizes to the peri-deuterosomal region and cooperates with PLK1 to mediate centriole release from the deuterosome (Fig. 2 C; Revinski et al., 2018). Despite the argument about the necessity of the deuterosome for centriole amplification in MCCs, the deuterosome-dependent pathway is extensively adopted in many multiciliated tissues and species. It will be important to fully elucidate the deuterosome composition and explore the role of the deuterosome during evolution.

How the centriole number is controlled in different types of MCCs remains unclear. The centriole number has been reported to be correlative with the surface area of MCCs in mouse trachea systems (Nanjundappa et al., 2019). The correlation between apical area and centriole number has also been confirmed in *Xenopus*, where the mechanosensitive cation channel PIEZO1 is essential for calibrating the centriole number in proportion to the apical area (Fig. 2 D; Kulkarni et al., 2021). In addition, the apical surface area expansion in mouse trachea MCCs requires appropriate centriole amplification. In PLK4- or CPAP-depleted MCCs, where centriole amplification is blocked, the apical surface area expansion is inhibited (LoMastro et al., 2022). Future studies are needed to explore thoroughly how centriole amplification and apical surface area expansion are mutually interconnected.

Interestingly, MCCs of *Xenopus* epidermis are specified in the basal epithelia and produce procentrioles as they migrate and intercalate into the outer epithelial layer, a process called MCC intercalation (Kulkarni et al., 2021). Their centrioles are produced in two waves: half of the centrioles are synthesized before intercalation and the remaining half are from the second round of centriole amplification during/after the apical expansion of the MCCs (Kulkarni et al., 2021). By contrast, similar radial intercalation does not occur and demanded centrioles are generated in a single round of amplification during the mammalian MCC differentiation (Al Jord et al., 2014; Yan et al., 2016). Therefore, it is worth exploring the details of centriole amplification during *Xenopus* MCC formation (the intercalation and apical expansion stages) by analyzing the role of these two centriole amplification pathways, which would provide additional information on the centriole amplification in different species.

#### Centriole dissociation and polarized migration

As procentrioles grow and form distal and subdistal appendages (Zhao et al., 2013, 2021), the matured centrioles start to dissociate from the parental centriole and deuterosome platforms, a process similar to the centriole disengagement during the centriole duplication cycle. Each newly assembled daughter centriole in cycling cells is tightly associated with its parental centriole through mitosis. As cells enter G1, the centriole pairs lose their tight configuration and disengage. Centriole disengagement is licensed by activation of the anaphase-promoting complex/cyclosome (APC/C) and mediated by separase and PLK1 (Tsou et al., 2009). CDK1-cyclin B phosphorylates SASS6, which successively disrupts its binding to STIL and further promotes cartwheel disassembly and centriole disengagement (Fig. 2 C; Huang et al., 2022). Interestingly, inhibition of the CDK1-APC/C or PLK1 in MCCs can lead to decelerated centriole dissociation from deuterosomes and trigger cell cycle re-entry (Al Jord et al., 2017; Krasinska and Fisher, 2018). Consistently, overexpression of EMI2, a potent inhibitor of the APC/C, also causes cell cycle re-entry in *Xenopus* MCC progenitors, similar to the effect of the APC/C inhibitor treatment in mouse brain MCC progenitors (Al Jord et al., 2017; Kim et al., 2022). However, depletion of EMI2 severely prohibits centriole dissociation from the parental centriole and deuterosome platforms (Fig. 2 C). The findings suggest that the activity of APC/C needs to be finely tuned for the regulation of centriole dynamics during MCC differentiation.

Additionally, in mouse and *Xenopus* MCCs, CDC20B located in deuterosomes is involved in separase-dependent centriole dissociation from deuterosome via the interaction with PLK1 (Revinski et al., 2018). In *Xenopus* MCCs, the dual specificity tyrosine phosphorylation regulated kinase 1A (DYRK1A) can phosphorylate CEP97, a centriolar distal end protein, which in turn recruits PLK1 and cooperates with separase to promote centriole dissociation (Lee et al., 2022). These studies further confirm that the activity of PLK1 and separase is indeed required for the centriole dissociation during MCC differentiation, indicating a general mechanism by which newly formed centrioles are separated from their nucleation platforms (Fig. 2 C). However, it remains unclear how PLK1 is activated and modulated during MCC differentiation.

Following centriole dissociation from the deuterosome, this structure is observed to break into pieces and the remnants lose the capacity to nucleate procentrioles (Kim et al., 2018; Zhao et al., 2013). In contrast to the deuterosome assembly process, the disassembly of the deuterosome remains poorly understood, although the signal that terminates the transcription of *Deup1* has started to be identified. CCNO is an MCC-specific cyclin, depletion of which in mice can result in severe hydrocephalus and mucus congestions in the respiratory tract (Funk et al., 2015; Núnez-Ollé et al., 2017). Further analysis of *Ccno*-deficient mice revealed that CCNO depletion inhibits the transcriptional downregulation of multiple factors for the deuterosome-dependent centriole amplification, including *Deup1*, *Cep152*, and *Mcidas*, which leads to an increase in deuterosome size (Funk et al., 2015). Interestingly, these enlarged deuterosomes in MCCs of *Ccno*-deficient mice remain intact even at the late stages of MCC differentiation (Funk et al., 2015), suggesting that CCNO is required for deuterosome disassembly or clearance. In addition, the EMI2-APC/C-PLK1 axis also functions in turning off the transcription of *Deup1* (Fig. 2 C; Kim et al., 2022). Future studies are needed to explore how CCNO and EMI2-APC/C-PLK1 mediate the on-off cycle of *Deup1* transcription for deuterosome disassembly. Testing whether posttranslational modifications regulate DEUP1 stability and degradation for deuterosome disassembly will be interesting.

Upon dissociation from the centriole nucleation platforms, centrioles migrate to the cell surface along the basal–apical axis, allowing centrioles to dock at the apical surface, a process dependent on various proteins and the cytoskeleton. The actin–myosin network is essential for centriole migration in MCCs as inhibition of actin polymerization or myosin function prevents centriole migration (Boisvieux-Ulrich et al., 1987, 1990). Consistently, in the ctenophores, the long-striated rootlet of newly formed centrioles in the cytoplasm closely associate with the parallel bundles of actin microfilaments oriented toward the cell surface (Tamm and Tamm, 1988). In support of the roles of the actin network in centriole apical migration, the small GTPases RHOA and CDC42 that directly regulate actin cytoskeleton dynamics are indeed required for directing centriole apical migration and docking in mouse trachea MCCs (Fig. 2 D; Pan et al., 2007). The Wnt/planar cell polarity (PCP) signaling pathway is essential for the apical positioning of centrioles. The PCP signaling proteins Dishevelled (DVL), Inturned (INTU), Fuzzy (FUZ), and DAAM1 mediate the RHO GTPase activity to regulate the actin network for the polarized migration of centrioles in MCCs (Gray et al., 2009; Park et al., 2008; Yasunaga et al., 2015). In addition, the intraflagellar transport (IFT) machinery also mediates the directional centriole migration. Interestingly, many of the actin dynamics regulators and IFT components are distributed to the migrating centrioles in *Xenopus* epidermal MCCs (Fig. 2 D; Park et al., 2008; Zhao et al., 2022), suggesting that the migrating centriole itself coordinates actin assembly for its apical movement.

#### Centriole transformation and docking

Following centriole migration, the mature centriole integrates into the apical cytoskeleton network and ultimately converts itself to a BB to initiate ciliary axoneme outgrowth. The mature centriole and ciliary BB possess distal and subdistal appendages (also known as basal feet), which are critical for BB anchoring. Interestingly, each motile cilium in MCCs has a cone-like basal foot on the lateral side of the BB, different from the presence of nine symmetrical basal feet per primary cilium (Fewell and Dutcher, 2020). In primary ciliated cells, cortical actin clearing and ciliary membrane partitioning are essential for ciliogenesis (Jewett et al., 2021; Smith et al., 2020; Zhao et al., 2023a). However, in *Xenopus*, cortical actin polymerization and subsequent actin network formation are required for the apical MCC emergence and centriole migration (Fig. 2 D; Sedzinski et al., 2016). It thus remains unclear whether cortical actin clearing occurs before centriole docking during multiciliogenesis.

Similarly, whether the formation of ciliary vesicles is involved in multiciliogenesis as in primary ciliogenesis is elusive. During primary ciliogenesis, small distal appendage vesicles (DAVs) dock to the distal appendage of the mother centriole and converge to form the ciliary vesicle (CV), which is regulated by RAB small GTPases and other membrane trafficking and remodeling effectors (Shakya and Westlake, 2021; Zhao et al., 2023a). Although small vesicles have also been observed distributed around the centrioles in MCCs (Dirksen, 1971; Sorokin, 1968), no membrane trafficking and remodeling effectors have been proven critical for multiciliogenesis. In mouse trachea MCCs, the ablation of CBY1, a binding partner of the distal appendage protein CEP164, leads to defective DAV accumulation around the migrating centrioles in the cytoplasm and impaired multiciliogenesis (Fig. 2 D; Burke et al., 2014; Voronina et al., 2009), suggesting that the CV formation may also occur during multiciliogenesis.

In primary ciliation, following the CV formation at the mother centriole, a cap complex formed by CEP97 and CP110 is removed from the centriolar distal end regulated by a variety of centriole remodeling proteins including KIF24, MPP9, TTBK2, ENKD1, and EHD1 (Goetz et al., 2012; Huang et al., 2018; Kobayashi et al., 2011; Song et al., 2022; Xie et al., 2023), which in turn allows the axoneme microtubule to extend (Zhao et al., 2023a). However, the function of the centriolar cap complex in MCCs has yet to be fully demonstrated. In contrast to findings in cultured mammalian primary ciliated cells, depletion of CP110 in *Xenopus* prevents the apical transport of centrioles and causes sequential defective cilia formation (Walentek et al., 2016), consistent with the finding that cilia formation is compromised in *Cp110* null mice (Yadav et al., 2016). Interestingly, *Cp110* knockdown can restore the defective centriole maturation and docking induced by miR-34/449 deficiency in MCCs of mice and *Xenopus* (Song et al., 2014). Thus, these studies suggest that the involvement of CP110 in ciliogenesis is under more complicated regulation in the animal in vivo systems. Considering the fundamental roles of other centriole remodeling proteins in primary ciliation, the tissue-specific knockout mouse model is helpful to explore their contribution to the centriole-BB conversion during multiciliogenesis.

Following completion of the cap complex removal, the transition zone, a region located at the proximal axoneme and adjacent to the BB, is assembled as the axoneme grows. In mammalian cells, CEP290 localizes to the centriolar satellites before ciliation and accumulates at the transition zone in ciliated cells (Tsang et al., 2008). The cap protein CP110 can interact with CEP290, an interaction essentially required for the ability of CP110 to suppress ciliogenesis (Tsang et al., 2008). Interestingly, the loss of CEP290 leads to sparse motile cilia in each ependymal MCC (Rachel et al., 2015), suggesting that CEP290 participates in multiciliogenesis. However, how CEP290 and its associated transition zone assembly are coupled with motile cilia formation awaits further exploration.

The actin cytoskeleton influences the apical centriole docking during multiciliogenesis. In *Xenopus*, WDR5, a core subunit of chromatin modifier, localizes to migrating centrioles and stabilizes the apical F-actin polymers for the apical MCC emergence and centriole docking (Kulkarni et al., 2018). Furthermore, a ciliary adhesion complex composed of focal adhesion proteins bridges the BB with the actin cytoskeleton, ensuring the BB anchoring (Antoniades et al., 2014). In *Xenopus*, the ciliary adhesion complexes are formed at the striated rootlet and the basal foot. The basal foot-located ciliary adhesion is assembled at the distal region of the basal foot and is required for the intact apical microtubule network (Chatzifrangkeskou and Skourides, 2022; Nguyen et al., 2020). On the other hand, the rootlet-located ciliary adhesion complex is linked to the apical actin filaments through the EZRIN-containing microridge actin-anchoring complex, which facilitates the interaction with the actin network (Walentek et al., 2016; Yasunaga et al., 2022).

In addition, FOXJ1 is implicated in centriole migration and docking in mouse and *Xenopus* MCCs (Brody et al., 2000; Gomperts et al., 2004; Stubbs et al., 2008; You et al., 2004). Defective centriole docking has also been observed in respiratory MCCs isolated from individuals carrying *FOXJ1* mutations (Wallmeier et al., 2019). FOXJ1 promotes the activity of RHOA to regulate the apical actin polymerization for proper centriole migration and docking (Pan et al., 2007). Gene ontology (GO) and enrichment analysis further suggest that the FOXJ1 regulatory network genes code not only proteins associated with ciliary structure and motility but also factors for the maintenance of epithelial cell polarity such as DLG4 and CDC42 (Mukherjee et al., 2019).

#### Assembly of multiple motile cilia

After docking to the plasma membrane, the A-tubule and its associated incomplete B-tubule of the centriolar microtubule triplet extend to form the axonemal doublet microtubule (DMT; Ishikawa, 2017). Similar to the centriolar microtubules, the α and β-tubulins of the DMTs undergo several conserved posttranslational modifications such as acetylation, glutamylation, and detyrosination, which contribute to the stability and function of cilia (Wloga et al., 2017). Meanwhile, the DMT-associating structures, including axonemal dynein complexes, nexin-dynein regulatory complexes (N-DRCs), and radial spokes (RSs), and axonemal inner central apparatuses (CAs) are assembled during the DMT growth. In this section, we describe the elemental composition of these motility-related structures and summarize how they are assembled and incorporated to form fully functional motile cilia.

**Figure 3.**
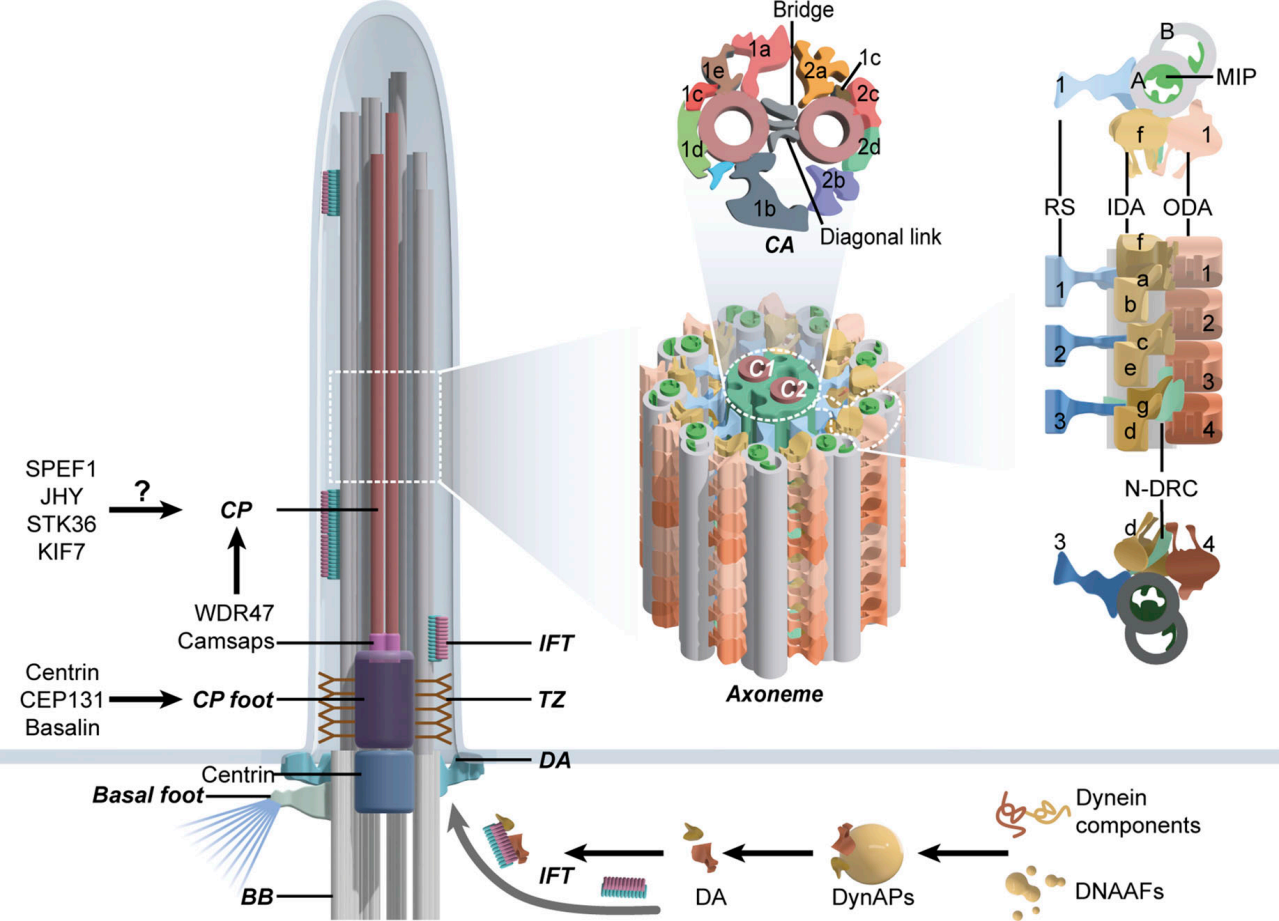
**Structure of the motile cilia axoneme.** A motile cilium comprises a basal body (BB), a transition zone (TZ), a centriolar microtubule extended axoneme, and a ciliary membrane. Compared with primary cilia, motile cilia display a 9+2 axonemal architecture with a central pair (CP) of microtubule-singlets (C1 and C2) surrounded by nine doublet microtubules (DMTs). The central apparatus (CA) distinguishes motile cilia from primary cilia, which includes the CP microtubules and their associating proteinaceous projections. The CP foot or basal plate is located at the proximal end of the CP and adheres to the distal end of the basal body, where WDR47 and Camsaps form a scaffold to nucleate the CP microtubules. The DMTs of motile cilia are decorated with T-shaped radial spokes (RSs), the nexin-dynein regulatory complexes (N-DRCs), and rows of axonemal dynein complexes (IDA and ODA). The dynein complexes are proposed to be preassembled in a cytoplasmic compartment (dynein assembly particle; DynAP) with the help of axonemal dynein assembly factors (DNAAFs) and transported into cilia via the IFT machinery. Structural biology studies reveal that a basic axonemal building block is 96 nm in length and each contains four ODAs (ODA1-4), one two-headed IDA (IDAf), six single-headed IDAs (IDAa/b/c/e/g/d), three RSs (RS1-3), and one N-DRC. These motility-related complexes may be anchored to the DMT through the interplay with microtubule inner proteins (MIPs).

To gain ciliary motility, axonemal dynein complexes (IDA and ODA) assembled on the A-tubule generate sliding force between two adjacent DMTs using ATP hydrolysis (Grotjahn and Lander, 2019). The N-DRCs that extend from the junction between the A and B- tubule of the same doublet and project across the inter-doublet space to connect with the B-tubule of the neighboring doublet transform inter-doublet sliding into axonemal bending (Heuser et al., 2009). In addition, T-shaped radial spokes protrude from each DMT and bridge with CAs for coordinating the ciliary motility (Grossman-Haham et al., 2021; Gui et al., 2021b; Zheng et al., 2021). Cryo-electron tomography (cryo-ET) and cryo-electron microscopy (cryo-EM) reveal that a basic axonemal building block is 96 nm in length and each block contains four ODAs (ODA1-4), one two-headed IDA (IDAf), six single-headed IDAs (IDAa/b/c/e/g/d), three RSs (RS1-3), and one N-DRC (Fig. 3; Osinka et al., 2019; Walton et al., 2023; Zimmermann et al., 2023). Recent cryo-EM studies further showed that an ODA docking complex links the ODA with the doublet microtubule inner protein (MIP) network (Fig. 3; Gui et al., 2021a, 2021b; Kubo et al., 2021; Walton et al., 2021).

A key question about multiciliogenesis is how those complex structures are properly assembled. To form all the motile cilia-specific structures mentioned above, hundreds of proteins need to be synthesized in a short period and cooperate to establish a precise and complicated arrangement. The IFT machinery is responsible for the assembly of primary cilia via active protein trafficking in and out of the cilia (Craft et al., 2015; Hao et al., 2011; Lechtreck, 2015; Nachury et al., 2010; Zimmermann et al., 2023). The IFT machinery comprises IFT-A and IFT-B complexes, which mediate anterograde and retrograde protein trafficking. The anterograde IFT delivers a variety of cargo molecules from the cell body to the ciliary tip and the retrograde IFT mediates turnover products from the tip to the cell body (Nakayama and Katoh, 2018). In mouse tracheal MCCs, IFT components are highly expressed during multiciliogenesis and the IFT transport also occurs along the axoneme of *Xenopus* epidermal motile cilia (Brooks and Wallingford, 2012, 2015; Xu et al., 2015), suggesting that the IFT machinery also mediates specific ciliary cargos for building the motile cilia of MCCs. Loss of IFT88, an essential component of the IFT machinery, indeed causes a reduced abundance of motile cilia in MCCs of mouse ependyma and trachea MCCs (Banizs et al., 2005; Gilley et al., 2014; Vladar and Stearns, 2007). Given that the IFT is essential for the establishment of the apical centriole migration (Cao et al., 2010; Zhao et al., 2022), the role of IFT in trafficking cargo for building motile cilia in MCCs however remains unclear.

Bardet-Biedl syndrome (BBS) proteins have been linked to the IFT machinery for transporting ciliary components (Nachury et al., 2007; Tian et al., 2023). Interestingly, BBS protein-deficient mice were observed to develop hydrocephalus progressively (Davis et al., 2007; Shah et al., 2008; Zhang et al., 2013). The loss of BBS proteins does not affect multiciliogenesis but causes the abnormal accumulation of vesicles in the ciliary shaft (Davis et al., 2007; Shah et al., 2008; Zhang et al., 2013), indicating that BBS proteins are dispensable for multiciliogenesis but required for the maintenance of ciliary membrane dynamics. In addition, some ciliary proteins or peptides have been proposed to be locally synthesized within the motile cilia of mouse ependymal MCCs. Inhibition of ciliary local translation resulted in motile cilia degeneration (Hao et al., 2021). This finding suggests that besides the IFT-mediated protein trafficking into the cilia, MCCs have adopted other strategies for simultaneously building multiple motile cilia.

Axonemal dynein arms are multiprotein complexes. An important aspect of axonemal dynein formation is the appropriate assembly of individual components synthesized in the cytoplasm. Given the huge amount and inherent complexities of axonemal dynein motors, many cytoplasmic factors such as RUVBL1, RUVBL2, WDR92, and ZMYND10, have been characterized as axonemal dynein assembly factors (DNAAFs), facilitating the preassembly of axonemal dynein arms in the cytoplasm (Fig. 3; King, 2021; Liu et al., 2019; Mali et al., 2018; Patel-King et al., 2019; Smith et al., 2022; Wang et al., 2022; Zur Lage et al., 2018). In vertebrate MCCs, the cytoplasmic axonemal dynein assembly was proposed to occur in the dynein assembly particle (DynAP), a cytoplasmic liquid-like compartment generated via phase separation of DNAAFs (Fig. 3; Horani et al., 2018; Huizar et al., 2018). Further analysis of this new MCC-specific organelle revealed that ODA and IDA are assembled in different subregions within DynAPs (Lee et al., 2020). This observation indicates dynein components are spatially restricted and this organelle is not in a liquid-like state. The concept of phase separation-mediated axonemal dynein assembly in MCCs thus needs further verification.

The presence of the central apparatus (CA) distinguishes motile cilia from primary cilia. The CA consists of a pair of central microtubules (C1 and C2) and their associating projections of interconnected complexes (Samsel et al., 2021). Unlike peripheral axonemal DMTs, the central pair (CP) microtubules do not originate from the BB. The observation that the minus-ends of CP microtubules attach a structure called basal plate or CP foot at the distal end of the transition zone (Dute and Kung, 1978; Gilula and Satir, 1972; Ringo, 1967; Zhao et al., 2021) raises a hypothesis that this structure may support the CP microtubule nucleation. Although trypanosome Basalin, *Chlamydomonas* Centrin and γ-Tubulin, and mouse Centrin and CEP131 have been located in this region (Fig. 3; Dean et al., 2019; Geimer and Melkonian, 2005; Silflow et al., 1999; Zhao et al., 2021), only the trypanosome Basalin depletion appears to cause the CA-loss defect (Dean et al., 2019). Thus, the role of the basal plate or CP foot in CA formation remains unclear. In mouse MCCs, WD40 repeat-containing protein 47 (WDR47) can enrich Camsaps, a family of microtubule minus-end binding proteins, at the proximal end of the CP microtubules (Buijs et al., 2021; Liu et al., 2021; Ren et al., 2022). Depletion of either WDR47 or CAMSAP3 causes the loss of CA in MCCs, resulting in symptoms reminiscent of primary ciliary dyskinesia in animals (Chen et al., 2020; Liu et al., 2021; Robinson et al., 2020; Saito et al., 2021). These findings suggest that the CP microtubules are nucleated on the scaffold formed by WDR47 and Camsaps in the proximal axonemal lumen (Fig. 3). In addition, several other proteins, including SPEF1, JHY, STK36, and KIF7, are essential for CA formation, but the detailed mechanism needs further exploration (Appelbe et al., 2013; Muniz-Talavera and Schmidt, 2017; Nozawa et al., 2013; Zheng et al., 2019). Moreover, the CP microtubules have numerous projections that share evolutionarily conserved structure and composition (Fig. 3; Han et al., 2022; Samsel et al., 2021). Although more CP-related proteins are being identified and characterized (Dai et al., 2020; Zhao et al., 2020b), how the functionally active CP projections are assembled as the CP microtubules grow in MCCs remains unclear.

In addition, RSs and N-DRCs must be precisely assembled during multiciliogenesis. As for the N-DRC, a recent study demonstrated the structural interdependency of the N-DRC components in mouse sperm flagella (Zhou et al., 2023), indicating a stepwise assembly of N-DRC via protein–protein interactions. By virtue of high-resolution cryo-ET and EM, structures of microtubule-bound RSs and N-DRCs are resolved (Gui et al., 2021b; Walton et al., 2023). In each periodic axonemal building block, RSs (RS1-3) are located to the docking adaptor formed by CCDC39-CCDC40 along the DMT with their head facing the CP projections (Gui et al., 2021b; Zheng et al., 2021), while the N-DRC occupies the junction between the bases of RS2 and RS3 in each periodic repeat, adjacent to several IDAs (Walton et al., 2023). The spatial interconnections of docking adaptors, RSs, IDAs, and N-DRCs indicate a complex assembly of these structures, which needs more comprehensive studies.

### Establishment of anatomically directed ciliary motility

**Figure 4.**
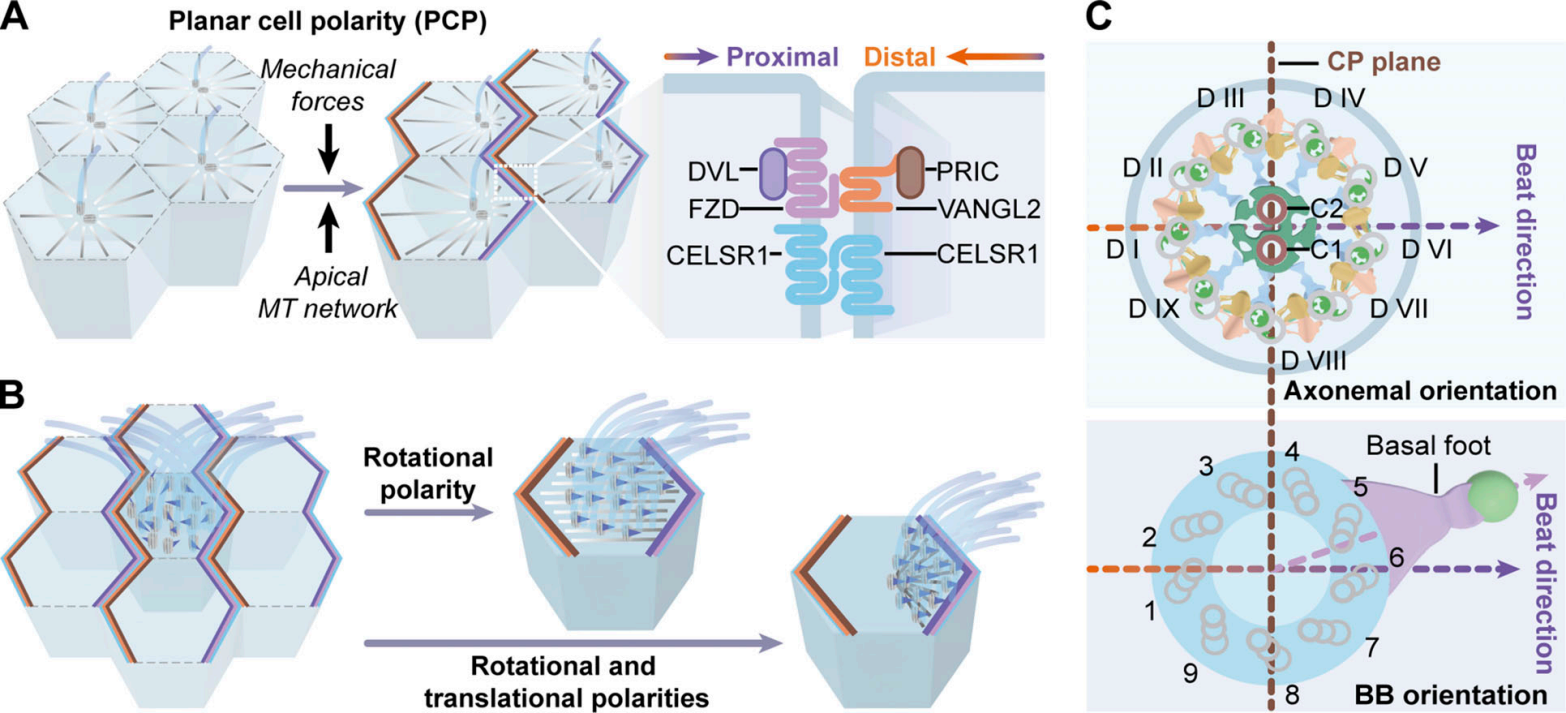
**Different types of polarities in multiciliated tissues. (A)** Planar cell polarity (PCP). The PCP is established before the formation of multiple motile cilia, a process relying on external mechanical forces and the apical microtubule network. Core PCP proteins such as Disheveled (DVL), Frizzled (FZD), Prickle (PRIC), and VANGL2 are asymmetrically distributed along the planar axis. The proximal (VANGL2 and PRIC) and distal (FZD and DVL) complexes segregate to opposite sides of the cell and interact with the opposite complex of the neighboring cell. CELSR1, a center component of the PCP system, is symmetrically distributed to both sides to stabilize the PCP complexes. **(B)** Rotational and translational polarities. The beat of newly developed motile cilia is in random directions and progressively aligned within each MCC (the rotational polarity). Different from the even distribution of basal bodies in MCCs of *Xenopus* epidermis and mammalian airway and reproductive tracts, basal bodies in matured ventricular MCCs are unidirectionally aligned within each cell (the rotational polarity) and uniquely clustered on one side of the apical surface (the translational polarity). **(C)** Axonemal and basal body (BB) orientations. Axonemal and BB orientations are used to assess the relationship between cilia movement and tissue axis. As shown, the nine ciliary doublet microtubules (DMTs) are numbered (D I-IX) and a unique structural feature exists between D V and D VI. The axonemal orientation is orthogonal to the CP plane defined by the line connecting the CP singlets, which runs through D I and across the space between D V and D VI. In MCCs, a cone-like basal foot is formed on the lateral side of the BB, which can occupy three of the nine triplet microtubules. The BB orientation is defined by the direction from the center of the BB to the tip of the basal foot. The axonemal orientation is aligned with the beat direction of motile cilia, while the BB orientation is aligned with the tissue axis.

The polarization of multiple motile cilia is essential for efficiently generating directional fluid flow over the epithelia of multiciliated organs (Fig. 4, A and B). Although newly developed motile cilia beat in random directions (Spassky and Meunier, 2017), the directions of cilia movements within each MCC are progressively aligned (the rotational polarity) and cilia movements of neighboring MCCs are also polarized along the tissue axis (tissue-level polarity; Fig. 4 B; Arata et al., 2022). Ciliary orientation is generally measured in terms of the BB orientation and the axonemal orientation, which are respectively defined by the direction from the center of the BB to the tip of the basal foot (BB orientation) and by the line connecting the CP microtubules (CP plane; Schneiter et al., 2021). Interestingly, the axonemal orientation is orthogonal to the CP plane and aligned with the beat direction of motile cilia, while the BB orientation is aligned with the tissue axis (Fig. 4 C; Schneiter et al., 2021). In addition, the distribution of BBs differs in MCCs originating from different tissues. In mammalian airway MCCs, BBs are evenly distributed throughout the entire apical surface, whereas BBs of mature ependymal MCCs are regionally clustered toward the cell–cell boundary along the plane polarity axis (the translational polarity; Fig. 4 B; Herawati et al., 2016; Mirzadeh et al., 2010; Vladar and Stearns, 2007). In this section, we will introduce how MCCs establish different polarities in ciliary beating.

PCP core proteins and downstream effectors coordinate to define and establish the tissue polarity axis. Interestingly, the PCP core proteins, including Frizzled (FZD), Disheveled (DVL), Prickle (PRIC), and VANGL2, are asymmetrically distributed along the planar axis with the proximal (VANGL2 and PRIC) and distal (FZD and DVL) complexes segregated to opposite sides of the cell, where they interact with the opposite complex of the neighboring cell (Fig. 4 A; Peng and Axelrod, 2012). This molecular asymmetry is established before cilia formation, which requires a polarized apical microtubule cytoskeleton (Fig. 4 A; Vladar et al., 2012). However, establishing intracellular rotational polarity is PCP-independent (Vladar et al., 2012). Loss of CELSR1 disrupts the asymmetry of PCP core proteins and the tissue-level BB alignment but has no effects on the intracellular polarized BB orientation in individual MCC (Boutin et al., 2014; Shi et al., 2014; Usami et al., 2021). By contrast, PCP signaling is essential for the tissue-level polarity (Guirao et al., 2010; Vladar et al., 2012). In ependymal MCCs of *Vangl2* knockout mice, although motile cilia can normally form and possess the beating ability, their BBs fail to establish the tissue-level alignment according to the external flow (Guirao et al., 2010).

Besides its role in the apical migration of centrioles, the actin network is also essential for coordinating cilia movement. In fully polarized MCCs, cytochalasin D treatment-induced actin depolymerization caused a disturbed BB alignment (Herawati et al., 2016; Werner et al., 2011). In addition, establishing BB orientation also requires the apical microtubule network. Treatment of MCCs with nocodazole leads to microtubule depolymerization and affects BB polarization (Herawati et al., 2016; Werner et al., 2011). In mice, loss of ODF2 disrupts the basal foot and the apical microtubule network, resulting in disorganized BB orientation in airway MCCs (Kunimoto et al., 2012). Furthermore, microtubule minus-end-binding proteins, γ-tubulin and CAMSAP3, have been shown to regulate the BB orientation via the apical microtubule network (Hagiwara et al., 2000; Robinson et al., 2020; Usami et al., 2021).

Previous studies have demonstrated that cilia motility and external hydrodynamic force contribute to the BB orientation (Fig. 4 B). In *Xenopus* MCCs, disruption of cilia motility resulted in disoriented BBs (Mitchell et al., 2007). In mice, loss of HYDIN or DNAAF2 impairs the motility of ependymal motile cilia and causes misoriented BBs (Lechtreck et al., 2008; Matsuo et al., 2013). Human nasal epithelial cells isolated from a patient bearing *GAS2L2* mutations consistently displayed defective cilia motility and severely randomized BB distribution (Bustamante-Marin et al., 2019). In addition, an external hydrodynamic effect also contributes to the unidirectional BB orientation. In *Xenopus* and mouse MCCs, the external fluid flow can redirect the BB orientation (Guirao et al., 2010; Mitchell et al., 2007). The alignment of BB can be more easily achieved in immature mouse ependymal MCCs at early stages than those at the later fully matured stage (Pellicciotta et al., 2020). Thus, the intracellular BB orientation requires proper cilia motility and external fluid flow, although the corresponding mechanism remains unclear.

### Multiple motile cilia-related ciliopathies

After years of scientific investigations, cilia have emerged as a critical cellular structure in organism development and homeostasis, dysfunctions of which can lead to a variety of genetic diseases, collectively referred to as ciliopathies (Fliegauf et al., 2007; Goetz and Anderson, 2010; Nigg and Raff, 2009; Zhao et al., 2023b). Given the confined distribution of multiple motile cilia in human organ systems, their defects may affect the airway, brain ventricular system, and male and female reproductive systems. Consistently, failure of motile cilia causes a genetic disorder, primary ciliary dyskinesia (PCD). Clinical features of PCD include neonatal respiratory distress, chronic sinopulmonary infection, laterality defects, infertility, and hydrocephalus in rare cases (Hyland and Brody, 2021; Wallmeier et al., 2020).

In the airway, motile cilia lining the respiratory tracts beat in a coordinated manner to facilitate the clearance of inhaled pathogens and particles by propelling mucus continuously from the lower airway. Abnormalities in the structure or function of the airway cilia impair mucociliary clearance and lead to chronic respiratory infections. In neonates, defective airway motile cilia impair mucociliary clearance during the transition from fetal to neonatal life, resulting in atelectasis and lobar collapse and causing neonatal respiratory distress (Machogu and Gaston, 2021). Consistent with the functional importance of axonemal dyneins in motile cilia, mutations of *DNAH5* and *DNAI1* together account for about 40% of PCD patients (Kurkowiak et al., 2015).

Multiple motile cilia in the brain ependyma facilitate the flow of cerebrospinal fluid, which is crucial for cell communication and neuron homeostasis (Kompaníková and Bryja, 2022). Disturbance of CSF flow by motile cilia impairment can cause seizure disorders, developmental delay, and hydrocephalus. While genetic mouse models of PCD frequently develop hydrocephalus, patients with PCD hydrocephalus are sporadic, except those with mutations in *FOXJ1*, *CCNO*, *P73*, and *MCIDAS* (Duy et al., 2022), indicating developmental differences between rodents and humans.

The multiple motile cilia in the efferent ducts of the testes beat in a whip-like rotary motion to prevent sperm aggregation (Rosenfeld, 2019). Defective motile cilia in efferent ducts of *GEMC1*, *MCIDAS*, or *CCNO* knockout mice cause rete testis dilation, sperm accumulation in the efferent ducts, and sperm missing in the epididymis, resulting in male infertility (Terré et al., 2019). Consistently, a male patient with *MCIDAS* mutations is infertile due to the loss of multiple motile cilia in the efferent ducts (Ma et al., 2021). In contrast, the fertility of females seems less affected, although multiple motile cilia exist in the fallopian of female reproductive tracts (Newman et al., 2023). A recent study using a genetic mouse model revealed that only motile cilia in the infundibulum are essential for female fertility but not those in other parts of the oviduct (Yuan et al., 2021). The fertility outcomes in women with PCD showed that about 40% of female patients reported natural pregnancy, much lower than the 90% in the general population, but the percentage of ectopic pregnancies in PCD women is similar to the general population (Newman et al., 2023), indicating an important role of multiple motile cilia in the reproductive ducts.

### Conclusion

In the past decade, great progress has been achieved in identifying proteins and related networks required for multiciliogenesis, determining the high-resolution structures of multiple large macromolecular complexes in motile cilia, and understanding the complex mechanisms underlying the regulation of multiple motile cilia formation. Considering the complexity and diversity of motile cilia, it remains a challenge to fully illustrate the genetic and molecular basis of multiciliogenesis. With integrative structural biology and efficient genetic tools, we will be able to characterize more critical components of the process, address the mechanism of multiciliogenesis in detail, and understand their roles in disease occurrence and progression.

## Supplementary Material

Table S1 is a list of transcriptional regulators of multiciliogenesis.
