# Supplementary material for: Formation and function of multiciliated cells
Source: J Cell Biol. 2023 Nov 30;223(1):e202307150. doi: 10.1083/jcb.202307150 (PMC10689204; doi:10.1083/jcb.202307150)
Supplement: Table S1 — is a list of transcriptional regulators of multiciliogenesis. [file JCB_202307150_TableS1.docx]

**Table S1 List of transcriptional regulators of multiciliogenesis.**

| **NO** | **Protein Symbol** | **Protein** | **Functions** | **References** |
| --- | --- | --- | --- | --- |
| 1 | SOX2/3 | SRY-box transcription factor 2/3 | Transcription factor; Regulates stem cell maintenance during lung development | Liu et al., 2013;  Lee et al., 2012 |
| 2 | P63 | Tumor antigen 63 | Transcription factor; Regulates cell cycle and basal cell maintenance during lung development | Bilodeau et al., 2021 |
| 3 | GEMC1 | Geminin coiled-coil domain-containing protein 1 | Transcription regulator; Downstream effectors of Notch signaling; Acts in a complex with E2F transcription factors to regulate the expression of MCIDAS, FOXJ1, RFX2, and RFX3 | Zhou et al.，2015;  Kyrousi et al., 2015;  Terre et al., 2016;  Arbi et al., 2016;  Lu et al., 2019 |
| 4 | MCIDAS | Multicilin | Transcription regulator; Downstream effectors of GEMC1; Acts in a complex with E2F transcription factors to regulate the expression of CCNO, CDC20B, DEUP1, and C-MYB | Stubbs et al., 2012;  Ma et al., 2014; Kyrousi et al., 2015; Lu et al., 2019; |
| 5 | E2F4/5 | E2F transcription factor 4/5 | Transcription factors; Form protein complexes with MCIDAS or GEMC1 and regulate the transcription of key genes for multiciliogenesis | Danielian et al., 2007;  Ma et al., 2014;  Danielian et al., 2016; |
| 6 | ARID1A | AT-rich interactive domain-containing protein 1A | GEMC1 binding protein; Regulates the transcriptional activity of GEMC1 during MCC formation | Lewis et al.，2023 |
| 7 | BRD9 | Bromodomain-containing protein 9 | MCIDAS binding protein; Regulates the transcriptional activity of MCIDAS during MCC formation | Lewis et al.，2023 |
| 8 | C-MYB | Transcriptional activator Myb | Transcription regulator; Regulates centriole amplification and acts upstream of FOXJ1 | Tan et al., 2013;  Pan et al., 2014;  Lu et al., 2019 |
| 9 | P73 | Tumor antigen 73 | Transcription factor; Regulates the expression of motile cilia-related factors such as FOXJ1, RFX2, and RFX3 | Marshall et al., 2016;  Nemajerova et al., 2016;  Wildung et al., 2019 |
| 10 | FOXJ1 | Forkhead box protein J1 | Transcription factor; Regulates the apical centriole migration and the expression of motility-related genes | Brody et al., 2000;  You et al., 2004;  Stubbs et al., 2008;  Thomas et al., 2010; |
| 11 | LRRC6 | Leucine-rich repeat-containing protein 6 | Dynein axonemal assembly factor; Regulates the dynein arm assembly and the nuclear translocation of FOXJ1 | Kim et al.，2023 |
| 12 | RFX2/3 | Regulatory Factor X 2/3 | Transcription factor; regulates the expression of genes for ciliary transport and basal body anchoring | Thomas et al., 2010; Lemeille et al., 2020 |
